# The Pulse and Its Observation by Nurses*Lecture delivered to Nurses of Lincoln Hospital, Feb. 28, 1912.

**Published:** 1912-11

**Authors:** Louis Faugeres Bishop

**Affiliations:** Clinical Professor of Heart and Circulatory Diseases, Fordham University School of Medicine, New York City; Physician to the Lincoln Hospital


### The Pulse and Its Observation by Nurses*

By LOUIS FAUGERES BISHOP, A.M., M.D.
Clinical Professor of Heart and Circulatory Diseases, Fordham University School of Medicine, New York City; Physician to the Lincoln Hospital.

The pulse occupies a good deal of the attention of physicians and nurses but it does not always occupy as much of the real thought as it ought.

* Lecture delivered to Nurses of Lincoln Hospital, Feb. 28, 1912.

The pulse is the general name given to the wave motion that is imparted to the blood current by some movement on the part of the heart. If you throw a stone into a lake, a wave which you can see with your eye is started that spreads in every direction on the surface of the water; or if in the dark you had your hand in the water you could feel the wave as it passed. That is like the pulse—a wave motion. It is not caused by the passage of blood, just as the wave of the lake is not caused by the passage of water. It is the passage of an impulse from one part of a body of blood to another part. In other words, it is a form of motion and not a material thing.

That is well illustrated by taking the end of a piece of string attached to something at the other end, and when you shake the string the wave which the motion generates at one end of the string passes to the other, but the string itself does not move from place to place. It is in just the same place as before the wave passed along it.

The pulse is a wave motion in the blood, and has nothing to do with the flow of the blood itself, so the pulse is not the measure of the amount of blood that is flowing. It is the measure of the amount of wave motion that is imparted to the blood by the contractions of the heart.

Another proof of that is that you can compress the artery completely in the wrist so that the blood cannot flow through it, and yet you can feel the pulse just above where it is compressed, although of course, there is no blood flowing through the vessel.

Up to quite recent times, when we spoke of the pulse it was understood that we referred to the pulse that is felt at the wrist—in the radial artery, and we considered that as the principal pulse in the body, though, it might be felt at the radial artery, the temple, or at one of the arteries of the leg. In other words, the arterial pulse was the only pulse we considered.

Of late years, those who have paid most attention to heart trouble have come to regard the pulse in the veins of great importance. It is indeed of great value and has reference to the contractions of the auricle. You know the heart is divided into four chambers, two auricles and four ventricles.

The four chambers of the heart come pretty closely together so that in a picture it is not easy to separate them visibly.

The blood enters the auricle, which is separated from the ventricle by valves, and then it enters the ventricle and is pumped into the arteries (we are speaking of the left side of the heart.) The contraction of the ventricle causes the pulse in the arteries. The contraction of the right auricle in health causes a pulsation in the veins called the jugular pulse because it is generally observed in the jugular vein.

The jugular pulse is of a good deal of importance to nurses provided they have been taught to observe it. You know from your experience that in very sick heart patients and in some other very sick patients you see a very marked pulsation in the neck. That is often the jugular pulse, and it has a particular significance. It means ordinarily that the auricle is not working properly, and that is a serious matter to the heart.

I have been at great pains to explain to you the nature of the pulse—that it was a wave motion and not the motion of the blood itself. The blood, of course, is flowing toward the heart through the veins, and yet the venous pulse travels from the hearts up to the veins. It might puzzle you very much if you thought about it. There is great beating in the veins but the blood is flowing in a different direction. The wave from the stone travels up-stream against the current if you throw a stone into the water. In the same way the venous pulse travels upward from the auricle into the veins.

I have recently had printed some nurses' charts for my private practice, and I put at the top of the chart that the nurse should observe any pulsation at the neck because in very sick people the onset of pulsations in the neck means weakness of the auricle and the auricle is the part of the heart which has to do with the origin of the contractions and its regularity. It is like the sparking part of an automobile; if this gets out of order the engine sputters and does all kinds of things; and it is the same way with the heart when the auricle misbehaves.

The most irregular hearts we have to deal with in very sick people are hearts in which the auricle is paralyzed, that is, there is a trembling palsy of the auricle, and these are the cases in which there is this marked pulsation in the neck.

In health the blood flows into the auricle, then the auricle gives a little contraction and drives the blood into the ventricle; then the ventricle contracts and drives it onward. Also, in health the jugular pulse comes a little before the radial pulse so that you find the pulse in the veins a little before the pulse at the wrist because the auricle contracts and drives the blood into the ventricle and the ventricle rontracts and drives it into the circulation.

This is the natural way, but this little healthy contraction of the auricle does not make a pulse that is big enough to be noticed. If you look very, very closely in a good light at a patient sometimes you can see the natural wave or auricular contraction in the jugular vein, but ordinarily it is too slight to be noticed; we can only get it by careful measurements with apparatus. So that the natural jugular pulse is not observable by ordinary means.

In bad cases of heart disease, the auricle becomes paralyzed and dilated, and is poisoned and does not work at all, and in these cases the contraction of the heart sends a wave back into the veins which is the visible pulsation in the veins that we are so familiar with in very sick heart patients. Now that wave, of course, really has its origin in the ventricle. In other words, it sends a wave back through the paralyzed auricle into the veins, and in that case our tracing from the veins would be very much like our tracing from the wrist. In other words, we get what is known as the ventricular form of venous pulse. This may seem a little complicated, but it is the latest advance in the understanding of heart disease. It involves more than half of the very bad heart cases that we see. It is a thing that can be easily suspected by a very simple symptom—by this pulsation in the neck. It is one of the great discoveries of modern times, so I do not hesitate to tell you about it.

When you put your hand on the pulse of a very sick patient and can see a pulse in the veins, which corresponds to the pulse at the wrist, particularly when the heart is irregular and rapid, you have every reason to suspect that there is paralysis of the auricle.

There is a very nice thing about this form of heart disease: that it yields to digitalis in a very specific manner. Digitalis often comes about as near bringing about the resurrection of a patient practically dead as any drug that I know. It restores the circulation, removes the dropsy, and gives the patient back a heart that is useful and able to carry on the circulation.

So I want you to remember that the pulse is a wave motion. It is not a material thing but a form of motion that is transmitted along the blood current, and has nothing to do with the movement of the blood as a whole.

I want you to remember that the pulse in the arteries is a wave motion and that it has its origin in the contractions of the ventricle or pumping part of the heart.

I want you to know about the fact of the existence of a pulse in the veins, and that it naturally occurs a little before the pulse at the wrist. Then I want you to remember that particular form of venous pulse that is easily visible and which corresponds in time with the pulsation at the wrist because it has its origin, not in the auricle, but in the ventricle.

The Chinese are said to be a people whose practice of medicine is founded almost entirely upon the pulse. The patient in China sometimes goes to the physician and thrusts his hand through a curtain and the physician feels his pulse, makes a diagnosis and prescribes. In the Chinese books of medicine there are hundreds of different kinds of pulses described. It is very foolish to say that any great nation is entirely wrong in anything that is a large part of one of their arts. However, we cannot help believing that the Chinese are a little one-sided in paying so much attention to the pulse, but they have undoubtedly been able through thousands of years of observation to put into writing a whole lot about the pulse which we never have put into writing, for the pulse in a great measure is to be estimated as a matter of judgment.

You feel the patient's pulse, and you say, "His pulse is better today than it was yesterday." Now what do you mean? You mean that you think it represents a better condition of affairs, but you may be very much in the wrong. A weak pulse is not exactly a bad pulse; a strong pulse is not necessarily a good one. Everything depends upon the circumstances. It is better to be guided by certain rules that we can formulate than it is to be guided by our impressions.

I don't want to try to imitate the Chinese and describe five hundred different kinds of pulses for I probably could not do it, but I want you as nurses to remember some fundamental things which are self-evident but which are often forgotten.

In the first place do not be misled by an apparent weakness of the pulse or encouraged by an apparently strong pulse. A weak pulse in a person generally feeble who is lying low, without any demands on the circulation, may be a conservative pulse for that person because it does not use up any energy and gives the patient a chance to recover. So a weak pulse that is not rapid in a feeble person is not alarming and not to be treated as such. The thing to be considered when the pulse is weak is, not whether the pulse can be made stronger because the pulse is only a wave motion transmitted from the heart. The thing to be considered is whether the blood is circulating properly; that is determined by the effect on the essential organs of the body. When I am teaching students, I repeat over and over again the signs of circulatory failure. These are shortness of breath on account of the deficiency of the blood supply to the lungs, swelling and tenderness of the liver because the blood is not pumped out of the liver, and dropsy.

Therefore, if the patient can lie down in bed and breathes quietly, is not tender over the liver, and there is no dropsy, ninety-nine times out of a hundred that patient is not in any danger from circulatory failure, and there is nothing to worry about because the heart is not sending big waves along the blood vessels to make a pulse.

On the other hand, a patient may have a pulse which is very hard and bounding which seems to be very strong—easy to feel and see and count—but that patient may be short of breath, have congestion of the liver and dropsy, and is in danger of circulatory failure and needs to be attended to.

Nurses often make the mistake of applying stimulation to weak patients to make the pulse stronger to feel, and it is a very foolish thing. It is not done so much in medical work as in surgical work. The foolish stimulation of the patient does him harm, and really wears out the heart and disturbs the circulation, making the chances of getting a real circulatory failure greater.

So remember that as long as the patient breathes well, has no congestion, and the blood is circulating properly, you need not worry about the character of the pulse.

There is one sign of circulatory failure that is of more importance than any other, and which the nurses should always watch, and that is, an increase in the rapidity of the pulse. If you will examine the charts of any one of fifty patients who have died with exhausting diseases or acute diseases, you will find that the failure of the heart was often characterized by an increase in rapidity. So that if the pulse averages 80 one day, 82 the next, 84 the next, 90-100-110-115-120--130-140-160—if the heart is becoming progressively rapid, that is always a very serious matter and should receive the utmost attention.

In considering this increase in rapidity, you have always to discount the presence of fever, because the two things that make the heart rapid usually are fever or weakness of the heart, so that if there is no increase in fever and the heart becomes gradually rapid, it is a serious sign.

Then it is particularly important in these cases, where we are paying so much attention to irregularities, to note any irregularity of the pulse, and describe it as best you can. Ordinarily irregularities are of two kinds: The lost beat and the extra beat. You will get a good deal of interest in examining your patients and making up your mind which is which.

"Lost beat" means that one of the beats got lost somewhere between the auricle and ventricle very often, or else that it did not originate at all. Now that is not the ordinary form of irregularity.

The ordinary form is the extra beat.

We have a beat, and then a little beat, then a long pause, and then a very big beat, and then a regular pulse for a while.

This is the ordinary form of irregularity which exists in a large number of people. I daresay if we could feel the pulses of all of you nurses we would find at least one person who once in a while had an irregularity of that kind. It is very common; it is not a lost beat at all; it is a little beat that comes too soon. It uses up the energy of the heart so that there is an extra long pause, then an extra big beat, and then the heart goes on regularly. This is called an extra systole. If you feel very lightly you can feel the little beat sometimes You feel many regular beats, then a little beat, and then a long pause; that is extra systole. There is apparently a beat lost because the beat is so small that it is often overlooked.

These are the two ordinary forms of irregularity. The form of which I have just spoken is of no particular importance. People have it who live to be a hundred years old and it does not affect them.

The lost beat is generally of some importance. We find it in people whose hearts are giving out. If you notice the heart of such a patient, you notice that you get lost beats. The heart fails to contract and the beats are dropped out. Of course, that is very often a serious matter. This may mean a heart that has lost its excitability and cannot respond. So the lost beat is important, though when you feel the pulses they may feel just alike.

In feeling a pulse you must not be misled by the difference in pulses which can be accounted for by the difference in the character of the vessel itself. In young people the vessel is very soft, and in old people you know the vessel is very hard. In young people the blood pressure is low, and in older people it is high. As you have often seen, and as I have just said, an old person may show signs of failure of the circulation, and yet have a strong pulse; a young person may have no signs of failure of the circulation with a weak pulse, so you have to discount this fact.

Always remember the importance of the circulation. If the patient's circulation is carried on badly, he always has shortness of breath, dropsy or congestion of the blood somewhere. Try to remember the distinction between the pulse and the circulation.

The different kinds of pulses are not worth detailed discussion. You have all felt the pulse of aortic regurgitation where the pulse is like pieces of shot rolling under your finger, the pulse going up and down very quickly.

You know the character of the pulse where there is mitral stenosis, where the quantity of blood that is circulating is very much diminished because of obstruction in the heart. You know the compressible pulse of fever patients, and in pneumonia you know how deceptive the pulse is because the pulse at the wrist may be very good, and yet the pulmonary circulation may be very near to failure.

The lesson I would like you to carry with you is not to draw too definite a conclusion from your own opinion as to the quality of the pulse—whether it is good or bad. Try to record and remember definite things, particularly the increasing rapidity not accounted for by fever. Watch for signs of circulatory failure and never neglect them; any patient short of breath is worthy a great deal of attention.

The counting of the pulse is a thing worth a good deal of attention, and is a matter that will often surprise you. I am surprised sometimes to this day by a "quick slow pulse" or a "slow quick pulse." Some pulses give the impression of a much greater rapidity than they have, and other pulses on account of the character seem to be rapid when we actually count that they are slow. So the only criterion is the actual count.

Another point is that if the pulse at the wrist is not actually felt, as often occurs because the radial happens to be deep, it is just as readily taken at the temple or in any accessible artery, because the blood wave passes through the body in a small fraction of a second and you can count the pulse where you please.

It is a good plan to be careful because I have noticed in a great many cases the pulse is recorded wrong for the reason that the nurse has only recorded the pulse as she felt it at the wrist. Now in irregular pulses what we want is the actual number of beats. We do not want only the big beats. We want all the beats as nearly as possible. I would so much rather see the report, "The pulse is not easily counted," or, "The pulse cannot be counted," or, "The number of appreciable beats is so and so," than a record of the big beats only. It is better in describing anything to describe it in full as you see it and not use too much condensation. It is always better to waste a few words in describing anything than it is to try to squeeze unusual facts into a conventional description of things.

54 West 55th Street, New York City, N. Y.

